# Daily fiber supplementation after bariatric surgery demonstrates tolerability and influence on the fecal gut microbiome

**DOI:** 10.3389/fnut.2026.1912097

**Published:** 2026-08-19

**Authors:** Brandy Moser, Solange M. Saxby, Stephanie Lebby, Jennifer D. Letendre, Sarah Lange, Marissa A. Mendez, Thadeus L. Trus, Elizabeth Honigsberg, Sarah E. Billmeier, Diane Gilbert-Diamond, Maria Carlota Dao, Jennifer L. Meijer

**Affiliations:** 1 Department of Agriculture, Nutrition, and Food Systems, University of New Hampshire, Durham, NH, United States; 2 Department of Community and Family Medicine, Dartmouth Health, Lebanon, NH, United States; 3 Section of Obesity Medicine, Center for Digestive Health, Dartmouth Hitchcock Medical Center, Lebanon, NH, United States; 4 Section of Bariatric Surgery, Center for Digestive Health, Dartmouth Hitchcock Medical Center, Lebanon, NH, United States; 5 Department of Epidemiology, Geisel School of Medicine, Dartmouth College, Hanover, NH, United States; 6 Department of Medicine, Geisel School of Medicine, Dartmouth College, Hanover, NH, United States

**Keywords:** bariatric surgery, butyrate, fiber, gut microbiota, obesity, potato starch, short-chain fatty acids, supplementation

## Abstract

**Introduction:**

This study assessed the feasibility and tolerance of a 30-day resistant potato starch (RPS) supplement in patients who recently underwent bariatric surgery and its effect on microbial production of fecal short-chain fatty acids (SCFA).

**Methods:**

This clinical trial (ClinicalTrials.gov, identifier: NCT05653648), conducted at Dartmouth Hitchcock Medical Center, provided 30-days of RPS supplement (titration: 24 g for 15 days; 48 g for 15 days) starting 90-days post-op and assessed gastrointestinal symptoms, dietary intake (24 h recall), fecal SCFAs, and fecal gut microbiome (*n* = 30).

**Results:**

The RPS supplement was well tolerated by participants (77% achieving consumption goal), with modest, but significant increases in self-reported gas (*p* = 0.004) and abdominal rumbling (*p* = 0.047). It was associated with an increase in the *Bifidobacterium* genus (LDA score >4, *p* < 0.001) and specifically, an enrichment of *B. faecale/adolescentis* (*p* = 0.006), one of two known primary degraders of RPS. No changes were observed in the abundance of cross-feeders or in SCFA concentrations. Alpha diversity measures were significantly lower post-RPS, and there was no change in species richness.

**Discussion:**

This study demonstrated that a RPS supplement is tolerable in post-op bariatric patients and that the gut microbiome is responsive to fiber interventions. RPS increased the abundance of SCFA-producing bacteria but did not alter fecal SCFAs, likely due to competition among cross-feeders.

## 1 Introduction

Over the past three decades, the rate of adult obesity has rapidly increased, such that in New Hampshire (NH), United States of America (USA), 9.9% of adults had obesity in 1990 compared to 29.9% in 2020 (1). Bariatric surgery, including Roux-en-Y gastric bypass (RYGB) and laparoscopic sleeve gastrectomy (LSG), is an effective treatment for obesity, typically yielding 50% excess weight loss and reducing long-term mortality (2). Guidelines from the National Institutes of Health recommend that bariatric surgery be offered to individuals with a body mass index (BMI) > 40 kg/m^2^ or a BMI of 35 kg/m^2^ with obesity-related co-morbidities (such as diabetes), and an estimated 270,000 bariatric surgeries are conducted annually in the USA (3).

Despite the effectiveness of bariatric surgery for weight loss, nutritional deficiencies are of great concern due to a lack of stomach volume, reduced food intake, and malabsorption (4–6). Nutritional management after surgery involves a series of diets, transitioning from liquid to pureed to soft foods, with a return to solid foods within a couple of months. Post-operative (post-op) bariatric surgery guidelines prioritize protein intake to ensure sufficient nutrients for wound healing and muscle mass maintenance, due to common experiences of early satiety (7). Fiber can be re-incorporated after bariatric surgery; however, post-bariatric populations are largely under-consuming fiber, with studies showing consumption ranging from 8–22 g/day (8–12). The Dietary Guidelines for Americans (DGA) recommend 25–34 grams of fiber (14 g/1000 calories) daily for adults; however, no clear guidelines have been established for fiber intake after bariatric surgery (13). While gastrointestinal intolerance to fiber-rich foods is a potential rationale for the decrease in fiber intake post-bariatric surgery (10), Fernandes et al. demonstrated adherence and tolerance to a low-viscosity fiber supplement, 6 g of fructo-oligosaccharides (FOS), initiated at 30-days after bariatric surgery (14).

Bariatric surgery alters the gastrointestinal environment, including the gut microbiome (15). Gut microbiome modifications are most drastic during the first 3 months after surgery, due to anatomical changes and alterations to dietary intake (15). Shifts in microbial diversity and composition are typically maintained after 3 months, with some studies showing regression toward pre-surgical composition after 1 year post-op bariatric surgery (15). Bariatric surgery has mixed impacts on the gut microbiome; with positive impacts such as increased alpha diversity, and negative impacts such as reduced straight short-chain fatty acids (SCFAs) (16–19). We have previously demonstrated that bariatric surgery is associated with a 26% decrease in SCFAs from pre- to post-surgery (~4 months), including butyrate (17).

Studies have shown that the gut microbiome and SCFA levels are responsive using short-term dietary interventions in a healthy population (20, 21). Short-term fiber interventions, such as potato starch, maize, inulin, FOS, and tapioca, in healthy populations have had consistent impacts on gut microbial composition, with enrichment of SCFA-producing taxa, including those of the *Bifidobacterium* genus (22). Baxter et al. demonstrated that resistant potato starch (RPS) resulted in the greatest increase in total SCFAs (32% increase), including butyrate (20). RPS is both non-bulking and non-viscous, making it an ideal choice for supplementation in a post-bariatric surgery population because they are susceptible to early satiety. In addition, RPS is moderately fermentable with the potential of modifying the gut microbiome and SCFA profiles in a post-bariatric surgery population. Modeling our clinical trial on Baxter et al., the objective of our study was to assess the impact of a 30-day RPS supplement on fecal SCFA-producing bacteria abundance and SCFA concentrations in a population that recently underwent bariatric surgery.

## 2 Materials and methods

### 2.1 Study cohort

This clinical trial (ClinicalTrials.gov, identifier: NCT05653648) was conducted at Dartmouth Hitchcock Medical Center’s (DHMC) Bariatric Surgery Program, testing compliance and gastrointestinal tolerance of a 30-day dose of RPS in bariatric patients from February 2023 through February 2024. Patients were recruited during a standard-of-care visit with the Bariatric Surgery Program Team at DHMC in Lebanon, NH, approximately two months after bariatric surgery. Inclusion criteria were as follows: (1) adults, age ≥18 years; (2) bariatric surgery utilizing RYGB or LSG; (3) the first occurrence of bariatric surgery; and (4) surgery through DHMC. At a telehealth appointment approximately 2.5 months after bariatric surgery, patients were screened for the following exclusion criteria: (1) current weight ≥550 lbs. (due to capacity of scales); (2) inadequate nutrition per standard of care guidelines as demonstrated by protein intake <60 grams per day and fluid intake <48 fl oz. per day (self-report); (3) allergies to potato starch; and (4) inability to speak and/or write in English. The study was monitored by the Dartmouth Health Human Research Protection Program (IRB; STUDY02001775), and all participants (*n* = 30) provided written consent.

### 2.2 Research study design

All patients received standard clinical care for bariatric surgery and followed a post-bariatric surgery visit schedule, with a minimal number of visits planned at 1, 4, and 12 months, then yearly. Patient information was extracted from the electronic medical record, including surgery date, surgery weight, surgeon, type of bariatric surgery, condition at discharge, 1-month follow-up date, and 1-month follow-up weight. Demographic and clinical data were collected as determined by the Metabolic and Bariatric Surgery Accreditation and Quality Improvement Program (MBSAQIP) and the American Society for Metabolic and Bariatric Surgery (ASMBS) guidelines, including sociodemographic information such as sex, age, race, ethnicity, education, and income (23, 24).

**Figure 1:**
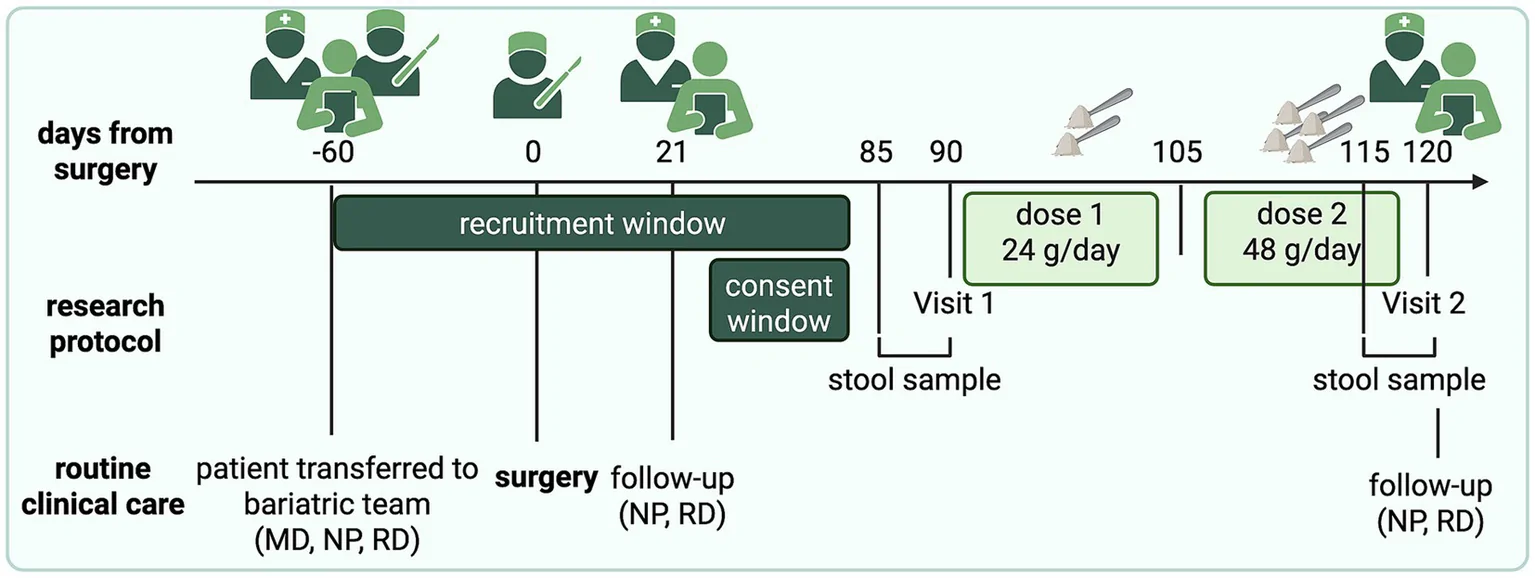
Timeline of research protocol and resistant potato starch dosing in comparison with routine clinical care. Created in BioRender. Meijer, J. (2026) https://BioRender.com/ed6m5n5.

The research protocol occurred between two clinical visits: Visit 1 (telehealth), taking place ~90 days post-surgery, and Visit 2 (telehealth or in-person clinic appointment), taking place ~120 days post-surgery (Figure 1). Study supplies were shipped to the participant prior to Visit 1, including two stool sample kits, 30 days of potato starch, a shaker, and a scale to measure body weight. Participants were asked to provide stool samples between 85 and 90 days post-surgery (pre-RPS) using the provided OMNIgene GUT kit (OMR-200 and ME-200 by DNA Genotek) and to return the sample to the research team via U. S. Mail. Samples collected are stable for several days at room temperature. Upon receipt at DHMC, samples were aliquoted and stored at −80 °C.

At Visit 1, participants completed 24-h dietary recall using the Nutritional Data System for Research (NDSR) The 24-h dietary recalls were used to assess diet food group consumption, as well as overall diet pattern using the, Health Eating Index (HEI) – 2015 score, which assess how closely a person’s dietary intake follows the DGA recommendations (25). During visit 1, an electronic survey disseminated using Research Electronic Data Capture (REDCap, RRID: SCR_003445), a secure, web-based software platform designed to support data capture for research studies, was administered regarding gastrointestinal symptoms, demographics, digestive history, and eating behaviors over the past 30 days (26). To evaluate gastrointestinal symptoms, the Gastric Symptom Rating Scale (GSRS) questionnaire was used (scored on a scale of 0–3, with three being most severe) (27, 28). The Three-Factor Eating Questionnaire (TFEQ)-R18 was used to evaluate eating behaviors among three sub-scores, with higher levels indicating higher perceived cognitive restraint, uncontrolled eating, and emotional eating (29). Participants were instructed to take a daily dose of 24 grams of potato starch (Bob’s Red Mill, Milwaukee, OR) administered via a shaker bottle mixed with a food or beverage for 15 days (Days 1–15) and complete a GSRS questionnaire on Day 3 of supplementation. On Day 16, participants were instructed to increase the dose to 48 grams daily (Days 16–30) and complete a GSRS questionnaire on Day 18. The potato starch contains approximately 70% resistant starch (type 2) by weight; therefore, subjects consumed approximately 17 grams and 34 grams of resistant starch, respectively (20). While taking the supplement, participants reported daily RPS intake and weighed themselves twice per week, with all metrics reported on a written log. Participants provided a second stool sample between 115 and 120 days post-surgery, which corresponds to day 26–30 of taking RPS dose 2 (post-RPS). Visit 2 occurred at the conclusion of the protocol and coincided with the 4-month standard of care follow-up visit. At Visit 2, participants completed an exploratory RPS preference survey (survey questions available in Supplementary Table 1) evaluating the taste and texture of RPS and the GSRS questionnaire to assess tolerance to the RPS supplement, as well as TFEQ questionnaires and a 24-h dietary recall using NDSR. Feasibility was defined as >70% of participants self-reporting ≥75% consumption of all RPS supplements and self-reported satisfaction during the RPS preference survey. Tolerance was classified as average GSRS scores ≤1, corresponding to occasional or no symptoms.

### 2.3 Short-chain fatty acid quantification

Targeted metabolomics was conducted by the Michigan Regional Comprehensive Metabolomics Resource Core (MRC2). Fecal samples were pre-weighed, homogenized via a probe sonicator, and centrifuged at 16000 rpm for 10 min. Supernatants were extracted with acetonitrile, and an internal standards mixture was used to create standard curves. The analytical platform was liquid chromatography/mass spectrometry (Agilent 6,410 QQQ) with mobile phase A: water + 0.1% formic acid and mobile phase B: methanol + 0.1% formic acid. Acetate, propionate, butyrate, valerate, caproate, hexanoate, isobutyrate, and isovalerate were profiled and normalized to account for the weight of feces.

### 2.4 Gut microbiota composition

Fecal samples were provided to the Marine Biological Laboratory in Woods Hole, MA, and the 16S rRNA V4 region was sequenced using the MiSeq. Demultiplexed FASTQ files were provided for further processing via QIIME2 (RRID: SCR_021258), DADA2 (RRID: SCR_023519) validated pipelines, using the SILVA 138.2 Ref NR 99 database for taxonomic classification (30). To avoid sequencing depth bias, Annotated amplicon sequencing variant (ASV) tables were rarefied to an even depth, and samples with < 20,000 reads were excluded prior to analysis. ASV tables were analyzed in RStudio using the phyloseq (RRID: SCR_013080) package for abundance analyses and quantification of microbiome diversity (alpha diversity, richness, and beta diversity) (31). Sequences of interest (SCFA-producing species, including primary and cross-feeders) were identified using the NCBI 16S rRNA sequence database (32). SCFA producers with identical V4 regions (i.e., *Bifidobacterium adolescentis, B. faecale,* and *B. stercoris*) were grouped as one species for the purposes of this analysis (20). Targeted analyses identified *R. bromii, B faecale/adolescentis, B. longum/breve, B. catenulatum/pseudocatenulatum/kashiwanohense, B. bifidum*, and *R. intestinalis* abundances. Microbial composition was compared pairwise pre- and post-RPS and correlated to fecal SCFA concentrations.

### 2.5 Statistical analysis

Paired t-tests (t.test) were used to identify differences in GI symptoms, dietary data, and TFEQ sub-scores by pre- and post-RPS. To identify differences in alpha diversity metrics after the intervention, paired Kruskal-Wallis tests were used. To assess differences in genus abundance after the RPS intervention, mixed linear models were utilized (MaAsLin3) with participant as the random effect (33). As a secondary analysis, a linear discriminant analysis (LEfSe) was performed to identify differences in bacteria (genus level) abundance according to pre- and post-RPS using the microeco package (34). A targeted analysis of SCFA species via NCBI BLAST (RRID: SCR_004870) was conducted among the top 500 prevalent ASVs, mirroring Baxter et al. (20). Pseudo counts of one were utilized in calculating fold changes of species abundance to account for zero inflated microbiome data. To assess increases in targeted taxa, paired, one-sided, Wilcoxon tests were conducted (wilcox.test). To determine if the levels of SCFA-producing bacteria abundance are associated with SCFA concentrations, Spearman correlation (rcor.test) and multivariable linear regression controlling for false discovery rate (FDR) were conducted. To determine if the potato starch intervention significantly altered levels of SCFAs, paired t-tests (t.test) and multivariable linear regression (lm) were conducted. For regressions, covariates that were considered include age, sex, weight at bariatric surgery, dietary starch, and fiber intake from 24-h recalls, and adherence to starch protocol. All statistical analyses were performed using R via RStudio 4.4.0.

## 3 Results

### 3.1 Population characteristics

Most participants were women (83%) with a self-reported white race (93%) and non-Hispanic ethnicity (97%). The median (Q1, Q3) age of participants was 47 years (39 years, 56 years). The majority of participants (77%) received a RYGB (Supplementary Table 2). The median pre-bariatric surgery weight was 272.6 lbs. (249.7, 306.2). Participants lost weight during the duration of the RPS, with a median 7 lbs. (5.1, 8.8) loss during the intervention (Supplementary Figure 1).

### 3.2 Potato starch intervention tolerance

**Figure 2:**
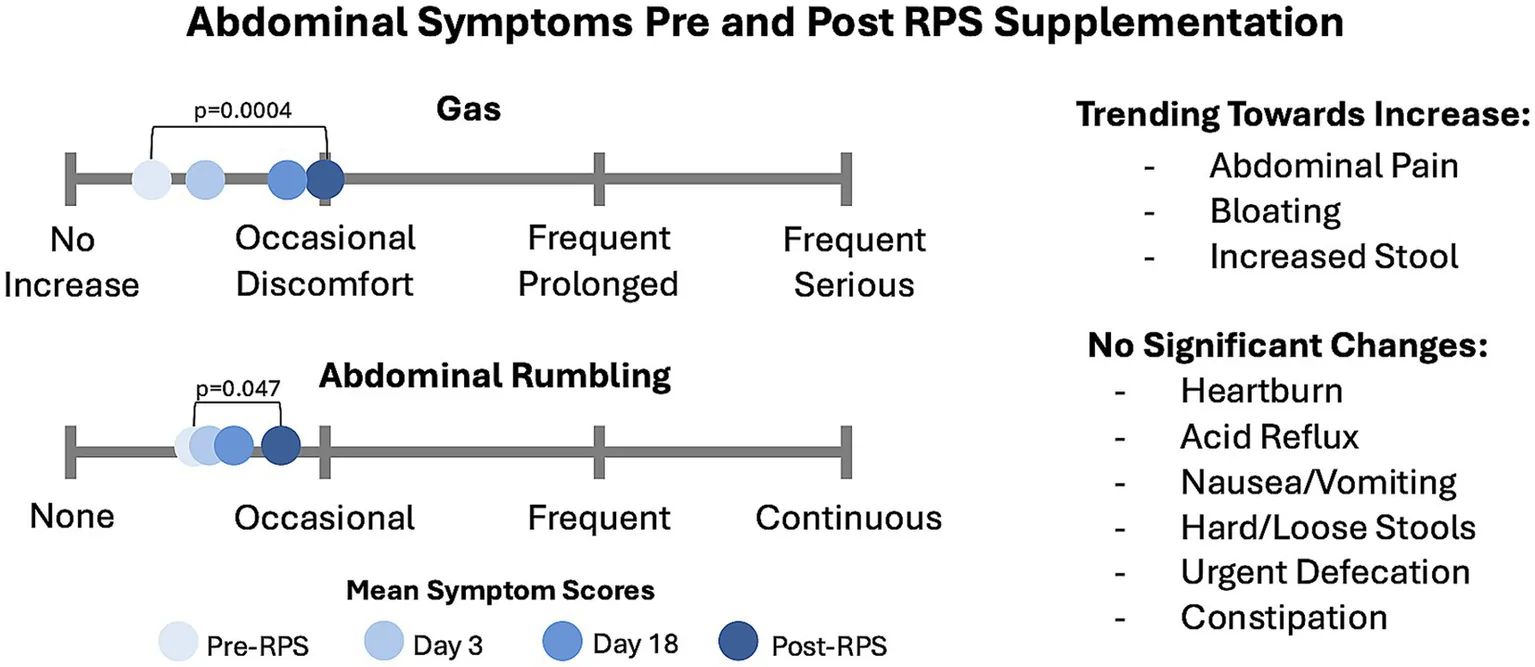
The Gastrointestinal Symptom Rating Scale, to assess supplement tolerance, was administered pre-resistant potato starch, on day 3, day 18, and post-resistant potato starch. Gas and abdominal rumbling symptoms increased significantly after 30 days of potato starch supplementation, as indicated by a paired *t*-test.

Overall, participants (*n* = 30) had an agreeable opinion of the RPS dissolvability, consistency, taste, and amount (Supplementary Table 1). Participants were neutral on their enjoyment of taking RPS (Supplementary Table 1). Notably, 77% of the cohort self-reported that they consumed ≥75% of all RPS supplements, and there was a trending association that participants who enjoyed taking RPS were more likely to have consumed a higher percentage of the RPS during the study (r = 0.36, *p*-value = 0.06). All average GSRS symptom scores were ≤1 pre- and post-bariatric surgery (data not shown). However, there was a significant self-reported increase in gas (*p* = 0.0004) in response to the RPS supplement, with average symptoms tracking from “normal” to “occasional discomfort for a short duration” (Figure 2). There was a significant self-reported increase in abdominal rumbling (*p* = 0.047) and a trend toward increases in abdominal pain (*p* = 0.083), bloating (*p* = 0.054), and increased stool frequency (*p* = 0.083). There were no differences in heartburn, acid reflux, nausea and vomiting, belching, stool consistency, urgent defecation, or constipation in response to the RPS supplement.

### 3.3 Dietary changes

There were no self-reported significant differences in protein, starch, or fiber intake in response to RPS supplement (Supplementary Table 3). A majority of participants did not meet DGA fiber recommendations (25–34 grams/day), with 100% of the participants pre-RPS (median: 7 grams) and 92% of the participants post-RPS consuming less than 25 grams of fiber daily (median: 10 grams) (13). Protein intake was greater than or equal to 60 grams (ASMBS guidelines) for 62% of the participants pre-RPS (median: 63 grams) and 73% of the participants post-RPS (median: 71 grams). There was no significant difference in Healthy Eating Index Score (HEI)-2015 in response to the potato starch supplement (25). Using TFEQ sub-scores, the overall population had moderately high cognitive restraint (median: 61.1) and low levels of uncontrolled (median: 3.7) or emotional eating (median: 5.6) at pre-RPS. TFEQ sub-scores were similar pre- and post-RPS intervention, with no significant eating behavior changes noted (Supplementary Table 3).

### 3.4 Microbiome changes

#### 3.4.1 Decreases in alpha diversity

**Figure 3:**
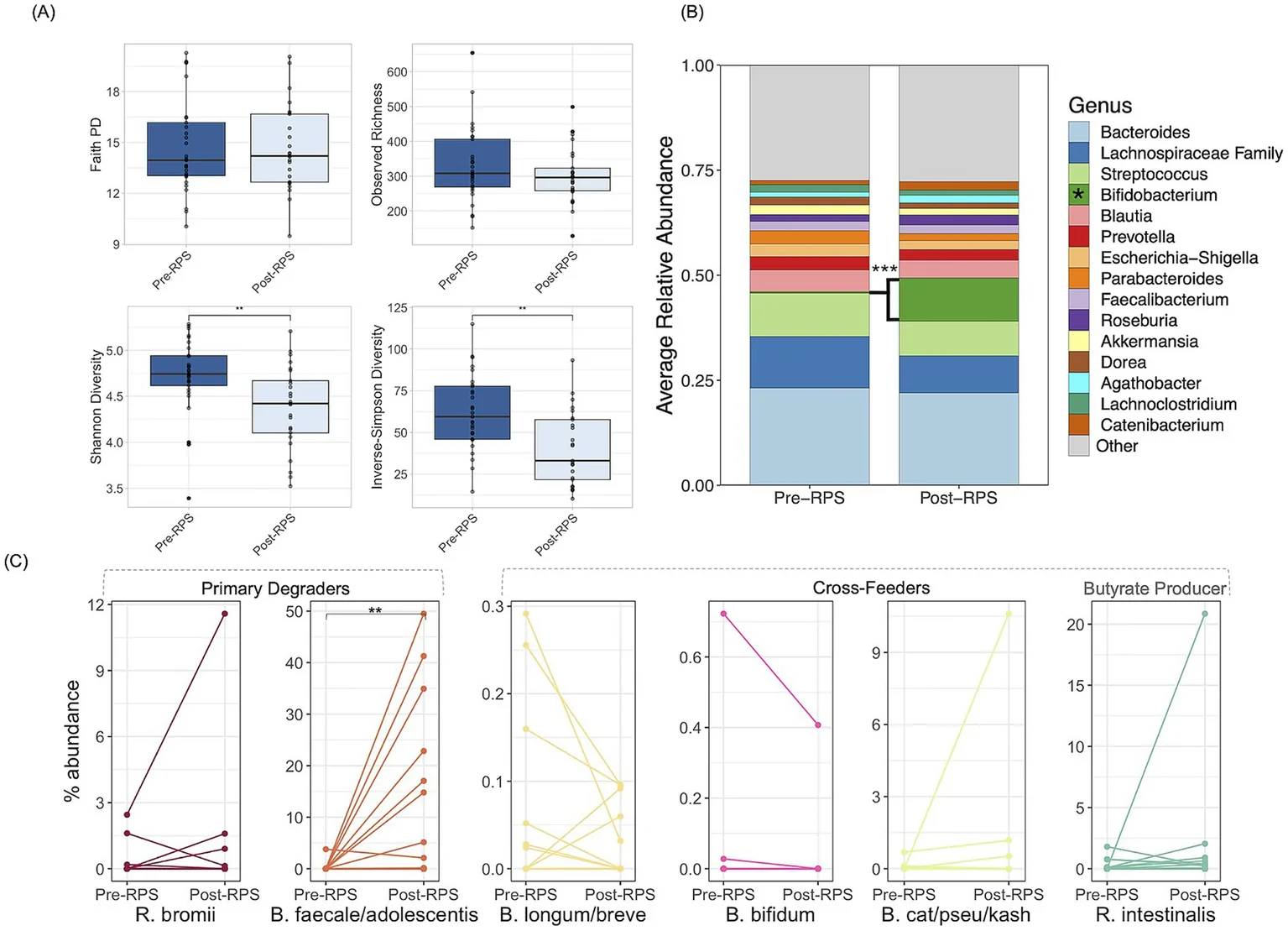
Changes in microbiome composition in response to resistant potato starch supplement. **(A)** Alpha diversity according to pre-resistant potato starch (Pre-RPS) and post-resistant potato starch (Post-RPS). There were no observed changes in Faith PD or observed richness. Shannon and Inverse-Simpson decreased significantly post-resistant potato starch (**, *p* < 0.01 by paired Wilcoxon test) **(B)** Average relative abundance of the top 15 most abundant genera according to pre- and post-resistant potato starch. *Bifidobacterium* was found significantly enriched post-resistant potato starch (***, *p* < 0.001 by untargeted linear discriminant analysis effect-sized). **(C)** Average relative abundance of resistant potato starch degraders and cross-feeders identified from targeted BLAST analysis of the top 500 most abundant ASVs pre- and post-resistant potato starch. *B. faecale*/*adolescentis* significantly increased post-resistant potato starch (**, *p* < 0.01 by one-sided paired Wilcoxon test).

Of the 30 participants, 26 had both pre- and post-stool samples, with two excluded due to low sample depth (*n* = 24). The alpha diversity metrics, Shannon (t-stat = 3.62, *p* = 0.001) and Inverse-Simpson (t-stat = 3.28, *p* = 0.003), were significantly lower post-RPS (Figure 3A). Observed richness (t-stat = 1.50, *p* = 0.15) and Faith PD (Kruskal-t-stat = −0.05, *p* = 0.96) were not significantly different, indicating observed decreases in alpha diversity could likely be attributed to a decrease in taxonomic evenness. Beta-diversity was not significantly different after RPS supplementation (*p* = 0.42) (Supplementary Figure 3).

#### 3.4.2 Enrichment in *Bifidobacterium* genus

*Bifidobacterium* was significantly enriched (*β* = 3.53, *p* = 0.0039) after RPS supplementation in a mixed linear model with participant as the random effect, however, significance was lost after BH correction. In a secondary analysis, LDA effect size (LEfSe) comparison of taxonomic abundance on the genus level also identified *Bifidobacterium* as significantly enriched post-RPS supplementation (*p* < 0.001), with observed increases in 18 out of 24 participants included in this analysis (Figure 3B; Supplementary Figure 4). No significant differences were observed in ASV relative abundance amongst the 500 most abundant ASVs. A targeted BLAST analysis identified specific SCFA-producing species. The percent abundance of *B. faecale/adolescentis* was significantly greater post-RPS supplement (V = 62, *p*-value = 0.006) (Figure 3C). *Bifidobacterium* was measurably increased post-RPS; however, it was not correlated to post-RPS SCFAs. Only valerate showed a significant negative correlation with *Anaerostipes* (R = −0.51, *p* = 0.049) abundance after post-RPS, after controlling for FDR.

#### 3.4.3 Classification according to *Bifidobacterium* response

Exploratory analysis classified participants as *Bifidobacterium* responders (% relative abundance change >0, *n* = 18) and non-responders (% relative abundance change ≤0, *n* = 6). There were no statistically significant differences in changes in SCFAs or percent change of SCFAs according to *Bifidobacterium* responder vs. non-responder status. *Bifidobacterium* responder status was also not significantly associated with surgery type or pre- and post-dietary data (protein, starch, fiber, and HEI). Furthermore, baseline microbiome composition was not related to *Bifidobacterium* responder status.

### 3.5 Changes in fecal SCFA

No changes in SCFA concentrations were observed pre- vs. post-RPS. Baseline SCFA concentration was not related to surgery type, sex, total fiber intake, or starch intake (data not shown). Only one SCFA, acetate, was correlated with pre-RPS weight (cor = 0.44, *p* = 0.02) (Table 1). No other SCFAs were related to any weight metrics.

**Table 1:** Short-chain fatty acids: pre- vs. post-resistant potato starch.

Short-chain fatty acid	Pre-resistant potato starch			Post-resistant potato starch			Percent change	Paired t-test	
	*n*	mean	median (Q1, Q3)	*n*	mean	median (Q1, Q3)	median % (Q1, Q3)	t-statistic	*p*-value
Acetate (nmol/mg)	30	51.65	46.5 (34.4, 59.5)	26	51.0	51.1 (35.5, 65.0)	9.15(−28.3, 57.5)	0.27	0.79
Propionate (nmol/mg)	30	14.88	13.9 (10.2, 17.3)	26	13.7	12.7 (7.9, 19.0)	−15.3(−44.4,41.8)	1.24	0.23
Isobutyrate (nmol/mg)	30	2.25	2.0 (1.4, 2.5)	26	1.8	1.6 (1.2, 2.1)	−16.6(−44.7, 24.7)	1.21	0.24
Butyrate (nmol/mg)	30	9.80	8.2 (6.9, 13.1)	26	11.3	8.8 (5.4, 14.6)	15.1(−33.4, 80.9)	−0.15	0.88
2-methylbutyrate, putative (nmol/mg)	30	0.84	0.7 (0.4, 1.1)	26	0.8	0.6 (0.4, 1.0)	−11.5(−50.7, 51.0)	0.094	0.93
Isovalerate (nmol/mg)	30	1.48	1.3 (0.9, 1.8)	26	1.3	1.0 (0.8, 1.4)	−15.1(−53.2, 68.9)	0.92	0.36
Valerate (nmol/mg)	30	2.77	2.0 (1.7, 3.3)	26	2.2	1.6 (1.0, 2.9)	−29.5(−59.0, 15.4)	0.89	0.38
Total SCFA (nmol/mg)	30	83.67	72.9 (60.7, 99.7)	26	82.01	81.3 (57.0, 100.2)	1.0(−27.1, 56.6)	0.38	0.71
Branched SCFA (nmol/mg)	30	3.73	3.2 (2.6, 4.45)	26	3.09	3.09 (2.0, 3.5)	−21.2(−50.2, 28.6)	1.27	0.22
Straight SCFA (nmol/mg)	30	79.94	70.5 (57.4, 92.6)	26	78.91	79.0 (54.3, 98.2)	4.47(−27.3, 54.9)	0.34	0.74

Paired t-tests were performed on log10 transformed SCFAs concentrations for *n* = 26.

## 4 Discussion

**Figure 4:**
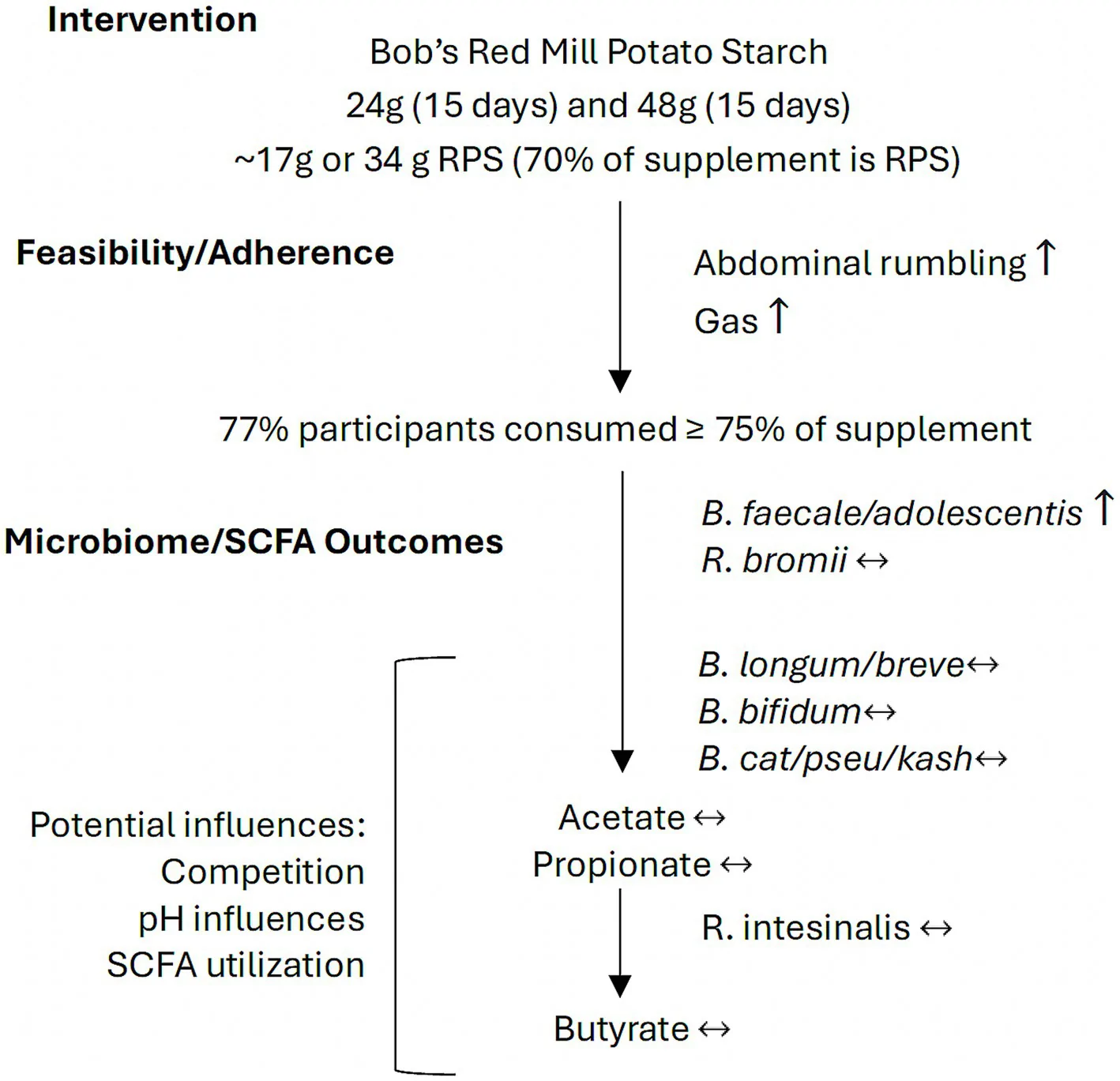
Flowchart of study intervention and key findings. The 30-day, two-step dose, resistant potato starch intervention (RPS) resulted in high adherence with some gastrointestinal side effects. The resistant potato starch intervention altered the microbiome, specifically increasing the abundance of primary degrader *B. adolescentis*. However, there were no changes in the identified cross-feeders or in short-chain fatty acids (acetate, butyrate, propionate).

We tested the feasibility and tolerance of a 30-day RPS supplement to alter fecal SCFAs and the abundance of SCFA-producing bacteria in a post-bariatric surgery population. RPS was selected as a low-viscosity fiber with the potential to alter the microbiome, and evidence suggests it could increase butyrate production (20). In the present study, RPS altered the gut microbiome, but did not impact fecal SCFA concentrations (Figure 4).

A significant decrease in diversity was observed in response to RPS supplementation. Diversity metrics, including Shannon and Simpson, combine measures of bacterial richness and evenness to characterize the composition of the gut microbiome (35). Lower gut microbial diversity is often an unfavorable outcome, but within fiber supplementation studies, it can be a sign that the intervention is effective at increasing the abundance of targeted bacteria (22, 36). There was no decrease in microbial richness or Faith PD, demonstrating that phylogenetic diversity, or the number of microbial members, was maintained, but rather the evenness of bacterial abundances was altered by RPS supplementation.

After the RPS intervention, we saw a notable increase in the genus *Bifidobacterium*, which has been previously demonstrated in other fiber interventions (RPS, inulin, and FOS) (22, 36). Furthermore, in a targeted analysis, we identified the *B. faecale/adolescentis* as significantly enriched post-RPS. We cannot distinguish *B. faecale* from *B. adolescentis* within this 16S rRNA analysis; however, *B. adolescentis* is a primary degrader of RPS and likely driving the observed increase in abundance. Primary degraders of RPS are pivotal, as they process RPS into accessible intermediates for cross-feeders to utilize. Currently, only two known primary degraders of RPS in humans have been identified: *R. bromii* and *B. adolescentis* (37) (Figure 4). Significant amounts of energy are directed to these primary degraders during a RPS intervention, often resulting in increased abundances (36). While *R. bromii* abundance increased in some participants post-RPS (change in % relative abundance >0, *n* = 5, Figure 3C), only increases in *B. faecale*/*adolescentis* were significant. *B. adolescentis* can produce a mixture of lactate, acetate, ethanol, and carbohydrates from RPS degradation, which are used by cross-feeders in butyrate production (37, 38). The primary degraders, *B. adolescentis* and *R. bromii,* are critical to initiating RPS breakdown, but their presence alone is insufficient for producing butyrate.

Cross-feeders utilize carbohydrates and acetate from the primary degrader RPS fermentation to further process into SCFAs. *R. intestinalis* was the only butyrate-producing cross-feeder identified in our targeted analysis, and its abundance did not significantly increase post-RPS. Our targeted analysis identified other cross-feeders, including *B. longum/breve*, *B. bifidum*, and *B. catenulatum/pseudocatenulatum/kashiwanohense*, which are actively competing for the same RPS breakdown products as *R. intestinalis* and producing other SCFAs like acetate, rather than butyrate (20). Our analysis did not observe any changes in the abundance of non-butyrate-producing cross-feeders, likely due to complex cross-feeding interactions, in which many bacteria are competing for the same resources (i.e., RPS breakdown products, lactate, acetate, ethanol) (38, 39). The spread of resources between many bacteria likely resulted in an unpredictable cross-feeder response and the lack of consistent changes in SCFA production. While there were likely other cross-feeders present, our study was limited to 16 s rRNA sequencing, and future studies would benefit from incorporating whole genome sequencing for improved species annotations. For example, *E. hallii* is a common low-abundance cross-feeder in humans but likely missed in our targeted analysis. *E. hallii* relies on lactate produced by *B. adolescentis* from RPS to produce butyrate (40).

Gathering results from Baxter et al. and our previous work, we hypothesized that RPS would increase butyrate production in post-op bariatric patients (17, 20). Our hypothesis was not supported by the results of this study, suggesting that dynamics may differ after bariatric surgery due to altered gastrointestinal conditions, microbial community relationships, or SCFA utilization. Additionally, Baxter et al. yielded increases in butyrate following a two-week RPS intervention in healthy adults, however, the 30-day RPS intervention duration may have been too short to detect measurable metabolic changes after bariatric surgery (20). pH has been shown to sway propionate and butyrate production and should be incorporated as a metric in future studies, given changes in gastrointestinal pH after bariatric surgery (41, 42). Nuanced microbial community relationships could have also influenced SCFA production. For example, increases in *R. bromii* (primary degrader) paired with *E. rectale* (cross-feeder) have been associated with a higher production of butyrate after the consumption of RPS (20). While our study assessed increases in specific bacteria, it did not consider the complex co-dependencies among microbial relationships that are needed to produce butyrate. Changes in SCFA utilization or absorption could be attributed to the lack of increased fecal SCFA levels, but remain difficult to monitor *in vivo* (18, 20, 38). Intestinal barrier function is often reduced in obesity and improves after bariatric surgery (43). Colonocytes that make up the intestinal barrier preferentially use butyrate as a fuel source; however, there has been no study investigating colonocyte usage of SCFAs after bariatric surgery or significant weight loss. Our findings suggest that while RPS is successful in altering *B. adolescentis* abundance post-op, it has unpredictable effects on cross-feeders and fecal SCFAs. Future directions include a randomized control trial with additional stool samples, pre-op bariatric surgery and post-RPS (45-day), to delineate the effects of RPS on the gut microbiome, SCFAs, and gastrointestinal symptoms (14).

Incorporating a RPS supplement has the potential to fill a dietary gap common in patients who have undergone bariatric surgery. Overall, many patients experience post-bariatric surgery increases in abdominal pain and constipation; therefore, patients reduce fiber intake for fear of exacerbating symptoms (27, 44). Within our study, the endpoint mean fiber intake (excluding RPS) was only 12 grams/day, less than half of the daily DGA recommendation (13, 45). Our study demonstrated that a RPS supplement could be feasible and tolerated post-op bariatric surgery following a two-step dosing regimen. While participants were neutral on the enjoyment of taking RPS, our study showed high adherence, indicating RPS supplementation is feasible post-bariatric surgery. Average gastrointestinal symptoms scores remained ≤1 (data not shown) throughout the supplementation and symptoms were particularly mild at the initial dose of 24 grams/day (20). However, gas and abdominal rumbling symptoms trended upward as the dose increased to 48 grams/day and increased significantly by the end of the study, after 30 days of RPS. Other studies suggest incorporating ~15 grams/day of RS for gastrointestinal and metabolic health improvements, and a dosage of 3.5 grams for 4 weeks was still shown to enrich *Bifidobacterium* in healthy adults (46, 47). Future studies may benefit from implementing a smaller dose or a slower dose buildup (e.g., starting at 25% of the goal dose and increasing every 2 weeks for an 8-week buildup) to ease GI symptoms (46, 48, 49).

Limited dietary intake and medication use data, in the absence of a control group, was a major limitation of the study. Our study incorporated only one pre-RPS and one post-RPS 24-h recall, which allowed for the quantification of short-term caloric, protein, starch, and fiber intake. Future studies should consider repeat 24-h recalls (2 weekday and 1 weekend) at multiple timepoints paired with a Food Frequency Questionnaire (FFQ) geared at accessing fiber intake and a quantification of fluid intake (50). After bariatric surgery, patients are instructed to avoid drinking during meals to avoid early satiety, making it challenging for patients to consume adequate amounts of fluid. Ensuring adequate fluid intake is necessary to avoid exacerbating GI symptoms from fiber intake. Medication use, particularly medications taken frequently after bariatric surgery including probiotics, proton pump inhibitors (PPI), laxatives, and vitamin supplementation, can alter the gut microbiome (51–54). We observed self-reported trends of decreased laxative use among a portion of participants (data not shown). Future studies should screen for antibiotic use as an exclusion criterion and consider quantitative metrics on stool softeners and laxatives, probiotics, PPIs, and vitamins.

To fill a dietary gap, RPS was selected for its non-bulking, non-viscous properties and its potential to modify the gut microbiome and increase SCFA production. RPS was successful in increasing the abundance of a SCFA producer, but did not yield changes in SCFAs, as seen in healthy populations (20). Future studies in bariatric surgery populations should utilize a control group to identify changes to the microbiome exclusively attributed to RPS, as natural temporal variation, dietary changes, medication use and post-surgical adaptations could have confounded current observations. Measures of pre-surgery microbiome composition, pH alterations, and host SCFA utilization in future research will clarify observed pathways. Additionally, pairing a smaller dose of RPS with other low-viscosity fibers, such as acacia gum, could provide a better opportunity for SCFA production, as a variety of fiber sources is often seen in habitual diets with higher fiber consumption (55). Currently, there are no studies identifying consistent increases in SCFAs from a particular combination of dietary fibers after bariatric surgery. Despite a lack of SCFA level increases, this study found that patient’s post-bariatric surgery could tolerate low-viscosity fiber supplementation and that the gut microbiome post-op is responsive to RPS interventions.

## Data Availability

The datasets presented in this study can be found in online repositories. The names of the repository/repositories and accession number(s) can be found at: https://www.ncbi.nlm.nih.gov/, PRJNA1474458.

## References

[1] Centers for Disease Control and Prevention (CDC) NC for HS (NCHS). National Health and Nutrition Examination Survey Data. Hyattsville, MD: Department of Health and Human Services, Centers for Disease Control and Prevention (2020).

[2] Kavanagh R Smith J Avgenackis E Jones D Nau P. A comparison of the effects of roux-en-Y gastric bypass and sleeve gastrectomy on body mass composition as measured by air displacement plethysmography. Obes Surg. (2020) 30:451–5. doi: 10.1007/s11695-019-04178-8, 31606840

[3] Estimate of Bariatric Surgery Numbers, 2011–2023. American Society for Metabolic and Bariatric Surgery [Internet]. (2025). Available online at: https://asmbs.org/resources/estimate-of-bariatric-surgery-numbers/ (Accessed November 18, 2025)

[4] Fujioka K. Follow-up of nutritional and metabolic problems after bariatric surgery. Diabetes Care. (2005) 28:481–4. doi: 10.2337/diacare.28.2.481, 15677821

[5] Handforth B Hennink M Schwartz MB. A qualitative study of nutrition-based initiatives at selected food banks in the feeding America network. J Acad Nutr Diet. (2013) 113:411–5. doi: 10.1016/j.jand.2012.11.001, 23438492

[6] Shikora SA Kim JJ Tarnoff ME. Nutrition and gastrointestinal complications of bariatric surgery. Nutr Clin Pract. (2007) 22:29–40. doi: 10.1177/011542650702200129, 17242452

[7] Hirsch KR Blue MNM Trexler ET Ahuja S Smith-Ryan AE. Provision of ready-to-drink protein following bariatric surgery: an evaluation of tolerability, body composition, and metabolic rate. Clin Nutr. (2021) 40:2319–27. doi: 10.1016/j.clnu.2020.10.022, 33158590

[8] Barstad LH Johnson LK Borgeraas H Hofsø D Svanevik M Småstuen MC . Changes in dietary intake, food tolerance, hedonic hunger, binge eating problems, and gastrointestinal symptoms after sleeve gastrectomy compared with after gastric bypass; 1-year results from the Oseberg study-a randomized controlled trial. Am J Clin Nutr. (2023) 117:586–98. doi: 10.1016/j.ajcnut.2022.11.016, 36811476

[9] Farias G Silva RMO da Silva PPP Vilela RM Bettini SC Dâmaso AR . Impact of dietary patterns according to NOVA food groups: 2 y after roux-en-Y gastric bypass surgery. Nutrition. (2020) 74:110746. doi: 10.1016/j.nut.2020.110746, 32200267

[10] Grosse CS Cope VC. Dietary fibre intake and bowel habits after bariatric surgery: a structured literature review. Obes Surg. (2019) 29:2247–54. doi: 10.1007/s11695-019-03837-0, 30903429

[11] Jeffreys RM Hrovat K Woo JG Schmidt M Inge TH Xanthakos SA. Dietary assessment of adolescents undergoing laparoscopic roux-en-Y gastric bypass surgery: macro- and micronutrient, fiber, and supplement intake. Surg Obes Relat Dis. (2012) 8:331–6. doi: 10.1016/j.soard.2011.11.016, 22260884 PMC3358526

[12] Novais PFS Rasera I Leite CV d S Marin FA de Oliveira MRM. Food intake in women two years or more after bariatric surgery meets adequate intake requirements. Nutr Res. (2012) 32:335–41. doi: 10.1016/j.nutres.2012.03.016, 22652372

[13] U.S. Department of Agriculture and U.S. Department of Health and Human Services Dietary Guidelines for Americans, 2020–2025. (2020). Available online at: https://www.dietaryguidelines.gov/sites/default/files/2020-12/Dietary_Guidelines_for_Americans_2020-2025.pdf (Accessed May 27, 2026)

[14] Fernandes R Beserra BTS Mocellin MC Kuntz MGF da Rosa JS de Miranda RCD . Effects of prebiotic and synbiotic supplementation on inflammatory markers and anthropometric indices after roux-en-Y gastric bypass: a randomized, triple-blind, placebo-controlled pilot study. J Clin Gastroenterol. (2016) 50:208–17. doi: 10.1097/MCG.0000000000000328, 25909598

[15] Santos JM Mathew MS Shah N Pajuelo-Vasquez R Mistry AM Heindl SE. Pre and post-operative alterations of the gastrointestinal microbiome following bariatric surgery. Cureus. (2021) 13:e13057. doi: 10.7759/cureus.13057, 33680599 PMC7929544

[16] Farup PG Valeur J. Changes in Faecal short-chain fatty acids after weight-loss interventions in subjects with morbid obesity. Nutrients. (2020) 12:802. doi: 10.3390/nu12030802, 32197409 PMC7146446

[17] Meijer JL Roderka MN Chinburg EL Renier TJ McClure AC Rothstein RI . Alterations in fecal short-chain fatty acids after bariatric surgery: relationship with dietary intake and weight loss. Nutrients. (2022) 14:4243. doi: 10.3390/nu14204243, 36296927 PMC9607039

[18] Seyfried F Phetcharaburanin J Glymenaki M Nordbeck A Hankir M Nicholson JK . Roux-en-Y gastric bypass surgery in Zucker rats induces bacterial and systemic metabolic changes independent of caloric restriction-induced weight loss. Gut Microbes. (2021) 13:1–20. doi: 10.1080/19490976.2021.1875108, 33535876 PMC7872092

[19] Shen N Caixàs A Ahlers M Patel K Gao Z Dutia R . Longitudinal changes of microbiome composition and microbial metabolomics after surgical weight loss in individuals with obesity. Surg Obes Relat Dis. (2019) 15:1367–73. doi: 10.1016/j.soard.2019.05.038, 31296445 PMC6722012

[20] Baxter NT Schmidt AW Venkataraman A Kim KS Waldron C Schmidt TM. Dynamics of human gut microbiota and short-chain fatty acids in response to dietary interventions with three fermentable fibers. MBio. (2019) 10:e02566-18. doi: 10.1128/mBio.02566-18, 30696735 PMC6355990

[21] Holmes ZC Villa MM Durand HK Jiang S Dallow EP Petrone BL . Microbiota responses to different prebiotics are conserved within individuals and associated with habitual fiber intake. Microbiome. (2022) 10:114. doi: 10.1186/s40168-022-01307-x, 35902900 PMC9336045

[22] Rodriguez CI Isobe K Martiny JBH. Short-term dietary fiber interventions produce consistent gut microbiome responses across studies. Res Sq. (2023). e00133–24. doi: 10.1128/msystems.00133-24, 38742890 PMC11237734

[23] Eisenberg D Shikora SA Aarts E Aminian A Angrisani L Cohen RV . 2022 American Society of Metabolic and Bariatric Surgery (ASMBS) and International Federation for the Surgery of obesity and metabolic disorders (IFSO) indications for metabolic and bariatric surgery. Obes Surg. (2023) 33:3–14. doi: 10.1007/s11695-022-06332-1, 36336720 PMC9834364

[24] Al-Mazrou AM Bellorin O Dakin G Pomp A Unruh MA Afaneh C. Implementation of the metabolic and bariatric surgery accreditation and quality improvement program and outcomes of bariatric surgery. Am J Surg. (2023) 225:362–6. doi: 10.1016/j.amjsurg.2022.09.059, 36208955

[25] Krebs-Smith SM Pannucci TE Subar AF Kirkpatrick SI Lerman JL Tooze JA . Update of the healthy eating index: HEI-2015. J Acad Nutr Diet. (2018) 118:1591–602. doi: 10.1016/j.jand.2018.05.021, 30146071 PMC6719291

[26] Harris PA Taylor R Thielke R Payne J Gonzalez N Conde JG. Research electronic data capture (REDCap)--a metadata-driven methodology and workflow process for providing translational research informatics support. J Biomed Inform. (2009) 42:377–81. doi: 10.1016/j.jbi.2008.08.010, 18929686 PMC2700030

[27] Høgestøl IK Chahal-Kummen M Eribe I Brunborg C Stubhaug A Hewitt S . Chronic abdominal pain and symptoms 5 years after gastric bypass for morbid obesity. Obes Surg. (2017) 27:1438–45. doi: 10.1007/s11695-016-2499-z, 28028658

[28] Svedlund J Sjödin I Dotevall G. GSRS--a clinical rating scale for gastrointestinal symptoms in patients with irritable bowel syndrome and peptic ulcer disease. Dig Dis Sci. (1988) 33:129–34. doi: 10.1007/BF01535722, 3123181

[29] de Lauzon B Romon M Deschamps V Lafay L Borys JM Karlsson J . The three-factor eating questionnaire-R18 is able to distinguish among different eating patterns in a general population. J Nutr. (2004) 134:2372–80. doi: 10.1093/jn/134.9.2372, 15333731

[30] Bolyen E Rideout JR Dillon MR Bokulich NA Abnet CC Al-Ghalith GA . Reproducible, interactive, scalable and extensible microbiome data science using QIIME 2. Nat Biotechnol. (2019) 37:852–7. doi: 10.1038/s41587-019-0209-9, 31341288 PMC7015180

[31] McMurdie PJ Holmes S. Phyloseq: an R package for reproducible interactive analysis and graphics of microbiome census data. PLoS One. (2013) 8:e61217. doi: 10.1371/journal.pone.0061217, 23630581 PMC3632530

[32] Camacho C Coulouris G Avagyan V Ma N Papadopoulos J Bealer K . BLAST+: architecture and applications. BMC Bioinformatics. (2009) 10:421. doi: 10.1186/1471-2105-10-421, 20003500 PMC2803857

[33] Nickols WA Kuntz T Shen J Maharjan S Mallick H Franzosa EA . MaAsLin 3: refining and extending generalized multivariable linear models for meta-omic association discovery. Nat Methods. (2026) 23:554–64. doi: 10.1038/s41592-025-02923-9, 41540124 PMC12982127

[34] Liu C Mansoldo FRP Li H Vermelho AB Zeng RJ Li X . A workflow for statistical analysis and visualization of microbiome omics data using the R microeco package. Nat Protoc. (2026) 21:1300–24. doi: 10.1038/s41596-025-01239-4, 40770112

[35] Cassol I Ibañez M Bustamante JP. Key features and guidelines for the application of microbial alpha diversity metrics. Sci Rep. (2025) 15:622. doi: 10.1038/s41598-024-77864-y, 39753610 PMC11698868

[36] Cantu-Jungles TM Hamaker BR. Tuning expectations to reality: Don’t expect increased gut microbiota diversity with dietary Fiber. J Nutr. (2023) 153:3156–63. doi: 10.1016/j.tjnut.2023.09.001, 37690780

[37] Jung DH Park CS. Resistant starch utilization by Bifidobacterium, the beneficial human gut bacteria. Food Sci Biotechnol. (2023) 32:441–52. doi: 10.1007/s10068-023-01253-w, 36911330 PMC9992497

[38] Teichmann J Cockburn DW. In vitro fermentation reveals changes in butyrate production dependent on resistant starch source and microbiome composition. Front Microbiol. (2021) 12:640253. doi: 10.3389/fmicb.2021.640253, 33995299 PMC8117019

[39] Cantu-Jungles TM Bulut N Chambry E Ruthes A Iacomini M Keshavarzian A . Dietary Fiber hierarchical specificity: the missing link for predictable and strong shifts in gut bacterial communities. MBio. (2021) 12:e0102821. doi: 10.1128/mbio.01028-21, 34182773 PMC8262931

[40] Belenguer A Duncan SH Calder AG Holtrop G Louis P Lobley GE . Two routes of metabolic cross-feeding between Bifidobacterium adolescentis and butyrate-producing anaerobes from the human gut. Appl Environ Microbiol. (2006) 72:3593–9. doi: 10.1128/AEM.72.5.3593-3599.2006, 16672507 PMC1472403

[41] Wang SP Rubio LA Duncan SH Donachie GE Holtrop G Lo G . Pivotal roles for pH, lactate, and lactate-utilizing bacteria in the stability of a human colonic microbial ecosystem. mSystems. (2020) 5:e00645-20. doi: 10.1128/mSystems.00645-20, 32900872 PMC7483512

[42] Reichardt N Vollmer M Holtrop G Farquharson FM Wefers D Bunzel M . Specific substrate-driven changes in human faecal microbiota composition contrast with functional redundancy in short-chain fatty acid production. ISME J. (2018) 12:610–22. doi: 10.1038/ismej.2017.196, 29192904 PMC5776475

[43] Jin Z Chen K Zhou Z Peng W Liu W. Roux-en-Y gastric bypass potentially improved intestinal permeability by regulating gut innate immunity in diet-induced obese mice. Sci Rep. (2021) 11:14894. doi: 10.1038/s41598-021-94094-8, 34290269 PMC8295358

[44] Kvehaugen AS Farup PG. Changes in gastrointestinal symptoms and food tolerance 6 months following weight loss surgery: associations with dietary changes, weight loss and the surgical procedure. BMC Obes. (2018) 5:29. doi: 10.1186/s40608-018-0206-4, 30524734 PMC6276242

[45] Dahl WJ Stewart ML. Position of the academy of nutrition and dietetics: health implications of dietary Fiber. J Acad Nutr Diet. (2015) 115:1861–70. doi: 10.1016/j.jand.2015.09.003, 26514720

[46] Bush JR Alfa MJ. Consumption of resistant potato starch produces changes in gut microbiota that correlate with improvements in abnormal bowel symptoms: a secondary analysis of a clinical trial. BMC Nutr. (2024) 10:152. doi: 10.1186/s40795-024-00962-7, 39605008 PMC11600726

[47] Miketinas DC Shankar K Maiya M Patterson MA. Usual dietary intake of resistant starch in US adults from NHANES 2015–2016. J Nutr. (2020) 150:2738–47. doi: 10.1093/jn/nxaa232, 32840627

[48] Al-Mana NM Robertson MD. Acute effect of resistant starch on food intake, appetite and satiety in overweight/obese males. Nutrients. (2018) 10:1993. doi: 10.3390/nu10121993, 30558330 PMC6316739

[49] Bush JR Baisley J Harding SV Alfa MJ. Consumption of SolnulTM resistant potato starch produces a prebiotic effect in a randomized, placebo-controlled clinical trial. Nutrients. (2023) 15:1582. doi: 10.3390/nu15071582 37049425 PMC10097138

[50] Yáñez F Soler Z Oliero M Xie Z Oyarzun I Serrano-Gómez G . Integrating dietary data into microbiome studies: a step forward for Nutri-Metaomics. Nutrients. (2021) 13:2978. doi: 10.3390/nu13092978, 34578856 PMC8468122

[51] Imhann F Bonder MJ Vila AV Fu J Mujagic Z Vork L . Proton pump inhibitors affect the gut microbiome. Gut. (2016). doi: 10.1136/gutjnl-2015-310376, PMC4853569 26657899

[52] Guetterman HM Huey SL Knight R Fox AM Mehta S Finkelstein JL. Vitamin B-12 and the gastrointestinal microbiome: a systematic review. Adv Nutr. (2021) 13:530–58. doi: 10.1093/advances/nmab123, 34612492 PMC8970816

[53] Vich Vila A Collij V Sanna S Sinha T Imhann F Bourgonje AR . Impact of commonly used drugs on the composition and metabolic function of the gut microbiota. Nat Commun. (2020) 11:362. doi: 10.1038/s41467-019-14177-z, 31953381 PMC6969170

[54] Afshar S Kelly SB Seymour K Woodcock S Werner AD Mathers JC. The effects of bariatric procedures on bowel habit. Obes Surg. (2016) 26:2348–54. doi: 10.1007/s11695-016-2100-9, 26894909 PMC5018031

[55] Rawi MH Abdullah A Ismail A Sarbini SR. Manipulation of gut microbiota using Acacia gum polysaccharide. ACS Omega. (2021) 6:17782–97. doi: 10.1021/acsomega.1c00302, 34308014 PMC8296006

[56] NIH conference. Gastrointestinal surgery for severe obesity. Consensus development conference panel. Ann Intern Med. (1991) 115:956–61. 1952493

