# Supplementary material for: Daily fiber supplementation after bariatric surgery demonstrates tolerability and influence on the fecal gut microbiome
Source: Front Nutr. 2026 Aug 19;13:1912097. doi: 10.3389/fnut.2026.1912097 (PMC13536892; doi:10.3389/fnut.2026.1912097)
Supplement: Supplementary file 1 [file Data_Sheet_1.pdf]

frontiers

Supplementary Material

1 Supplementary Figures and Tables

Supplementary Table 1. Evaluation of resistant potato starch preferences.
(1) strongly disagree, slider, (5) strongly agree

Continuous Variables	n	median (Q1, Q3)
The packages of the potato starch were easy to add to the shaker.	30	5 (4.25, 5)
The shaker helped to dissolve and mix the potato starch with a liquid.	30	5 (4, 5)
The texture of the potato starch mixed with a liquid or soft food was acceptable.	30	5 (3.25, 5)
The taste of the potato starch mixed with a liquid or soft food was acceptable.	30	4.5 (3, 5)
The amount of the potato starch in each bag was acceptable to take at one time.	30	5 (3.25, 5)
I enjoyed taking the potato starch supplement.	30	3 (2, 4)

Supplementary Table 2. Bariatric surgery type (n=30)

**Surgery Type**	**n**	**percent**
Roux-en-Y Gastric Bypass	23	77%
Laparoscopic Sleeve Gastrectomy	7	23%

Supplementary Table 3. Diet and eating behavior changes: pre-resistant potato starch vs. post-resistant potato starch. Grams of resistant potato starch were not included in dietary intake.

**Variables**	**Pre-RPS**			**Post-RPS**			**paired t-test**	
	n	mean	median (Q1, Q3)	n	mean	median (Q1, Q3)	t-statistic	p-value
Protein (g)	30	65	63 (54, 74)	26	73	71 (59, 85)	1.56	0.13
Carbohydrates (g)	30	55	53 (30, 65)	26	68	60 (41, 104)	1.45	0.16
Starch (g)	30	19	15 (6, 24)	26	26	18 (11, 43)	1.150	0.26
Fiber (g)	30	9	7 (4, 11)	26	12	10 (5, 17)	1.49	0.15
Total Energy (kcals)	30	759	732 (567, 912)	26	926	936 (628, 1152)	3.00	0.006
HEI-2015 score	30	55	55 (45, 61)	26	59	57 (46, 74)	0.796	0.43
**TFEQ Sub-Scores**								
Cognitive Restraint	27	56.2	61.1 (50.0, 66.7)	26	60.0	58.3 (50.0, 76.4)	0.665	0.51
Uncontrolled Eating	28	11.2	3.7 (0.0, 18.5)	28	11.1	7.4 (2.8, 18.5)	-0.570	0.57
Emotional Eating	28	12.3	5.6 (0.0, 22.2)	29	10.34	0.0 (0.0, 22.2)	-1.044	0.31

Healthy Eating Index (HEI), Three-Factor Eating Questionnaire (TFEQ)
TFEQ sub scores transformed to 0-100 scale

1.1 Supplementary Figures

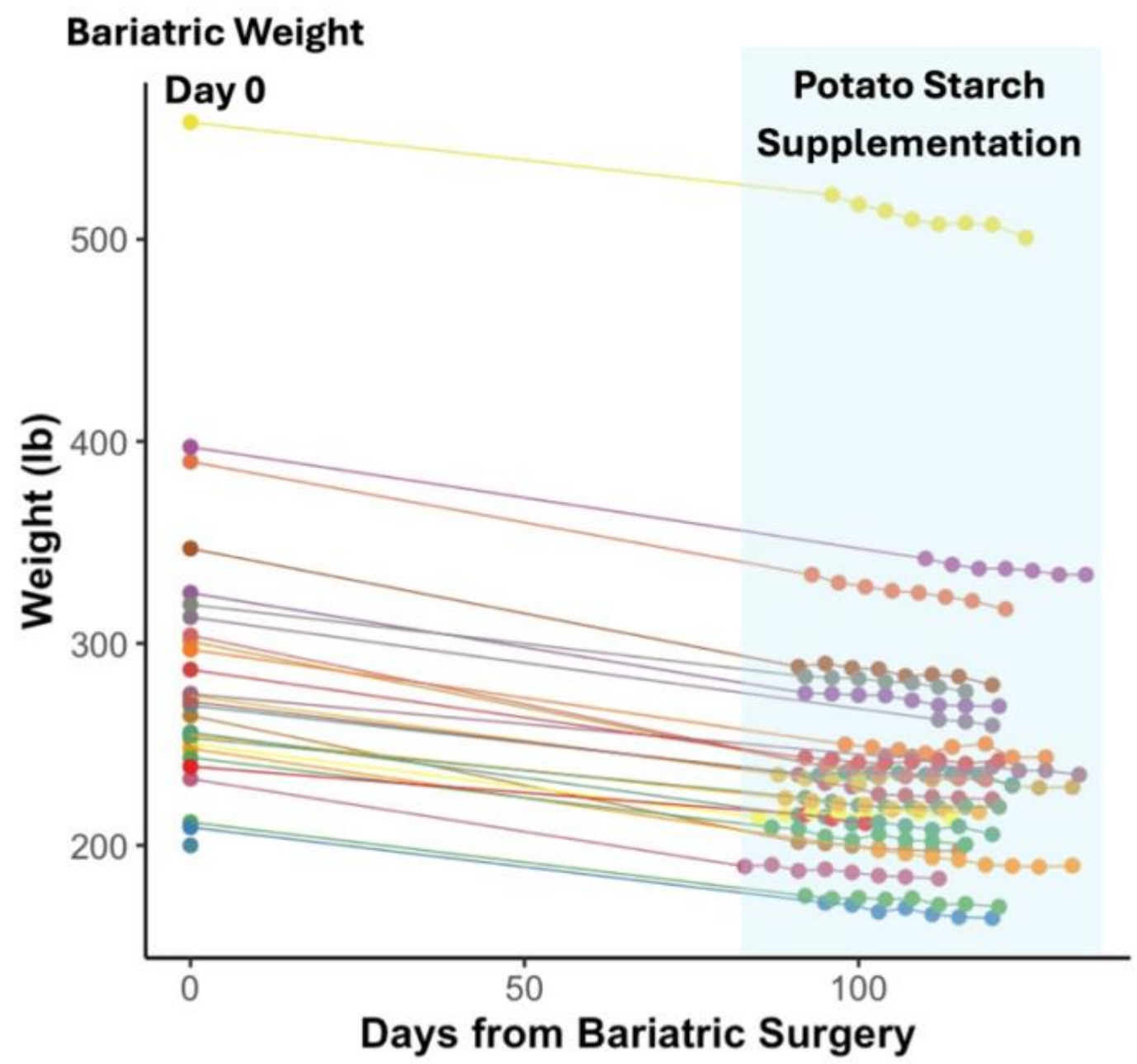

Supplementary Figure 1. Participant weight at bariatric surgery (day 0) and self-reported weights during resistant potato starch supplement.

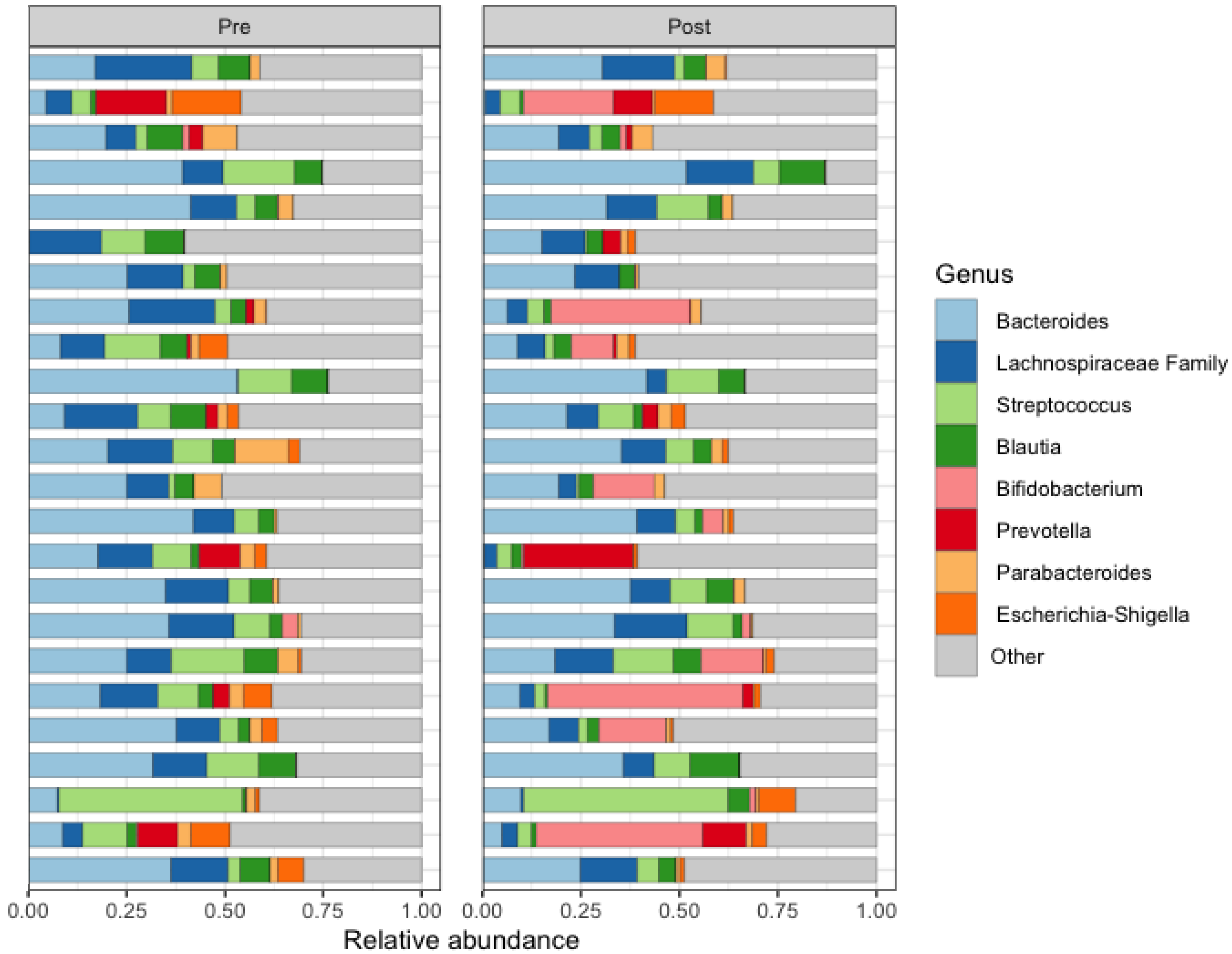

Supplementary Figure 2. Relative abundance of the top 8 most abundant genera by individual participant pre-resistant potato starch (Pre) and post-resistant potato starch (Post).

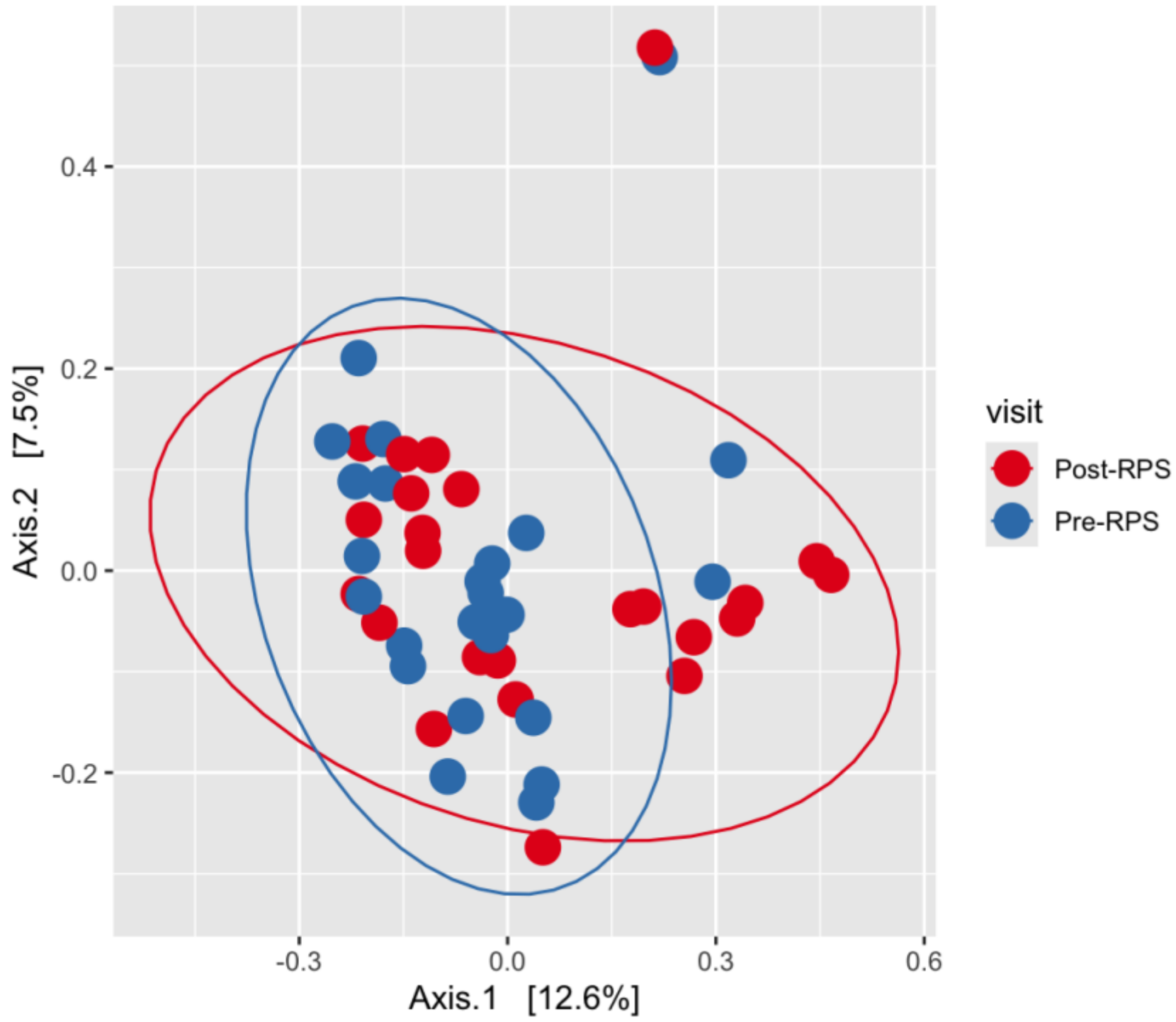

Supplementary Figure 3. Bray-Curtis beta diversity did not significantly differ according to pre-resistant potato starch (Pre-RPS) and post-resistant potato starch (Post-RPS) (p=0.42 by PERMANOVA).

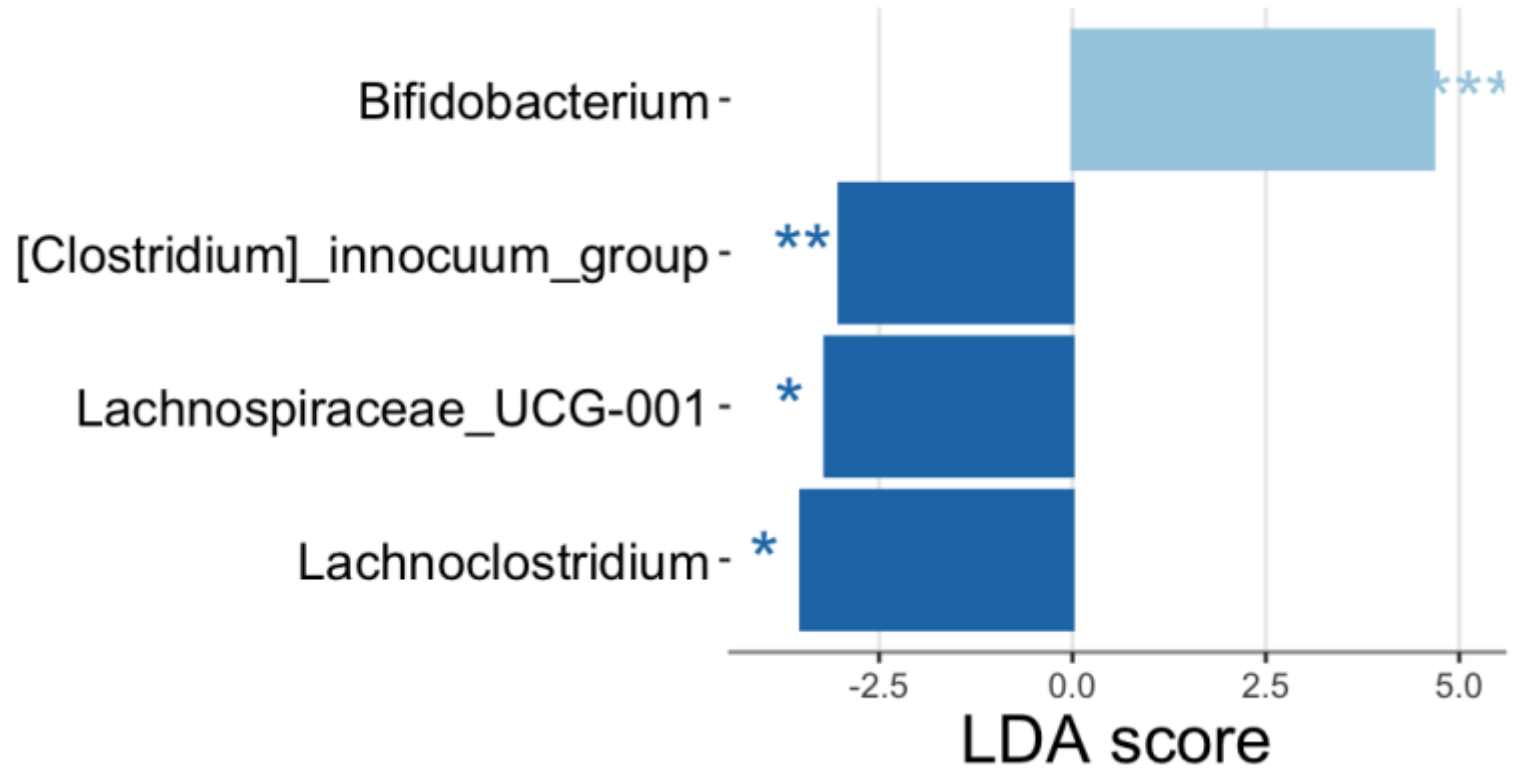

Supplementary Figure 4. Linear discriminant analysis scores of untargeted genus relative abundance relative to pre-resistant potato starch. *Bifidobacterium* was the only genus enriched post-resistant potato starch (***, $p<0.001$ by linear discriminant analysis effect size), while 3 genera of the Clostridium family were significantly depleted post-resistant potato starch (*, $p<0.05$ and **, $p<0.01$ by linear discriminant analysis effect size).
